# Neuroprotection in Preterm Infants

**DOI:** 10.1155/2015/257139

**Published:** 2015-01-11

**Authors:** R. Berger, S. Söder

**Affiliations:** Marienhaus Klinikum St. Elisabeth, Department of Obstetrics and Gynecology, 56564 Neuwied, Germany

## Abstract

Preterm infants born before the 30th week of pregnancy are especially at risk of perinatal brain damage which is usually a result of cerebral ischemia or an ascending intrauterine infection. Prevention of preterm birth and early intervention given signs of imminent intrauterine infection can reduce the incidence of perinatal cerebral injury. It has been shown that administering magnesium intravenously to women at imminent risk of a preterm birth leads to a significant reduction in the likelihood of the infant developing cerebral palsy and motor skill dysfunction. It has also been demonstrated that delayed clamping of the umbilical cord after birth reduces the rate of brain hemorrhage among preterm infants by up to 50%. In addition, mesenchymal stem cells seem to have significant neuroprotective potential in animal experiments, as they increase the rate of regeneration of the damaged cerebral area. Clinical tests of these types of therapeutic intervention measures appear to be imminent. In the last trimester of pregnancy, the serum concentrations of estradiol and progesterone increase significantly. Preterm infants are removed abruptly from this estradiol and progesterone rich environment. It has been demonstrated in animal experiments that estradiol and progesterone protect the immature brain from hypoxic-ischemic lesions. However, this neuroprotective strategy has unfortunately not yet been subject to sufficient clinical investigation.

## 1. Introduction

The prevention of preterm birth represents one of the most significant challenges to the field of obstetrics in the 21st century. Preterm infants born before the 30th week of pregnancy are especially at risk of prenatal mortality and morbidity [1]. Damage to the immature brain is one of the central concerns. Typical lesions include peri-/intraventricular hemorrhages (PIVH) and periventricular leukomalacia (PVL). Both of these complications specifically affect the pyramidal tracts of the lower extremities. The resulting damage leads to spastic cerebral palsy of the legs [2].

## 2. Peri-Intraventricular Hemorrhage and Periventricular Leukomalacia

**Figure 1:**
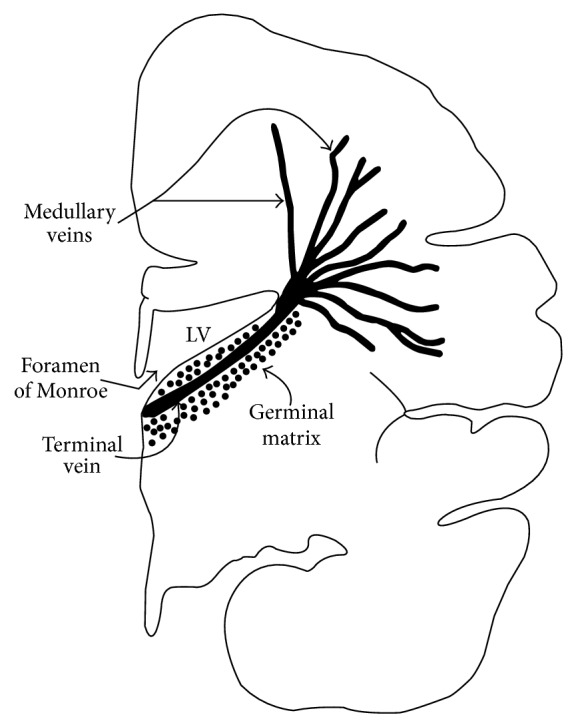
Germinal matrix, the predilection site for peri-intraventricular brain hemorrhage among immature fetuses [2].

PIVH originates in the vascular bed of the germinal matrix, an area of the brain that almost completely disappears as the fetus matures [3–5] (Figure 1). Blood vessels in this area of the brain burst very easily [6, 7]. Pre- and postpartal fluctuations of the cerebral blood flow can thus lead to the rupture of these blood vessels and induce PIVH [8–12]. The extent of the hemorrhage can be increased by an alteration in the thrombocyte aggregation and the coagulation system [13–15]. Such hemorrhages have been shown to lead to the destruction of the germinal matrix, periventricular hemorrhagic infarction of the white brain matter, and hydrocephalus [2].

**Figure 2:**
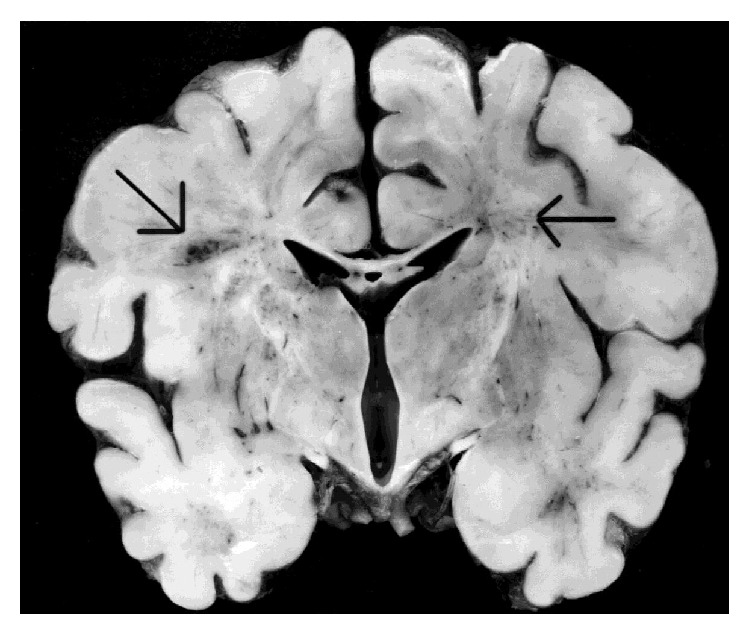
The left arrow marks an intraventricular hemorrhage (PIVH). The right arrow marks an area of periventricular leukomalacia (PVL) [25].

PVL most commonly leads to damage of the radiatio occipitalis on the trigonum of the lateral cerebral ventricles and the white matter around the foramen of Monroe [16, 17] (Figure 2). This involves axons and oligodendrocytes, especially those that are in the early stage of development. Activated microglia then enter the lesion and strip away the necrotic tissue. Subsequently, small cysts form, which can then be identified sonographically [18–20]. The lack of myelinisation as a result of damaged oligodendrocytes and an expansion of the lateral cerebral ventricle are then the consequence [21–24].

PVL can be caused by both cerebral ischemia and infection. During the genesis and the development of the cerebral vascular bed, vascular watersheds develop in the radiatio occipitalis on the trigonum of the lateral cerebral ventricles and the white matter around the foramen of Monroe [26–28]. The vasodilatation capacity and thus the ability to increase blood circulation during and after arterial hypotension appear to be very restricted in these areas of the brain [29]. After the 32nd week of pregnancy, the vascularisation of these predilection sites increases significantly and the likelihood of PVL decreases.

**Figure 3:**
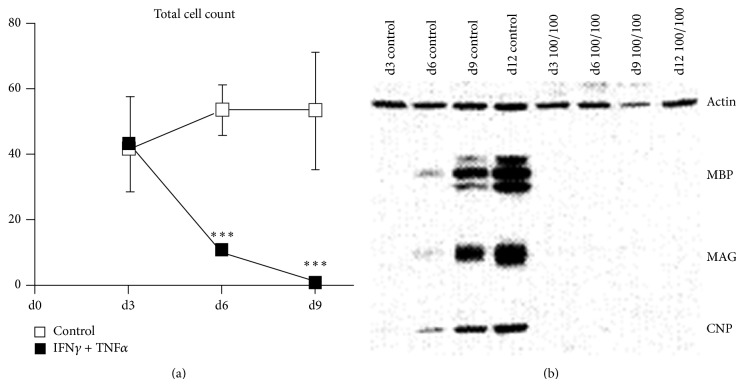
(a) Oligodendrocyte precursor cells between the 3rd and 9th day in culture (d3–d9). The* y*-axis shows the number of cells per field of vision. The administration of INF-*γ* (100 U/mL) and TNF-*α* (100 ng/mL) severely reduced the number of surviving cells (^***^ *P* < 0.001). Western blot was conducted for MBP, MAG, and CNP. On the 12th day in culture pronounced expression of MBP, MAG, and CNP was observed, indicating the differentiation of the oligodendrocyte precursor into the mature cell type. The administration of IFN-*γ* and TNF-*α* almost completely inhibits the expression of these proteins [38]. IFN-*γ* = interferon gamma; TNF-*α* = tumor necrosis factor-alpha; MBP = myelin-basic protein; MAG = myelin associated protein; CNP = 2′,3′-cyclic nucleotide 3′-phosphodiesterase.

Ascending intrauterine infections can also induce PVL [30–32]. An ascending infection causes a so-called “fetal inflammatory response syndrome” [33]. The release of endotoxins associated with this syndrome leads to serious impediment of the fetal cardiovascular system regulation, resulting in a reduction in cerebral blood circulation and thus in ischemic lesions in the white brain matter [34, 35]. Cytokines, glutamate, and free radicals are also able to directly damage oligodendrocytes in the early stages of development and thus also disrupt the subsequent myelinisation process, which can significantly affect the development of an infant's motor skills [36–38] (Figure 3).

**Figure 4:**
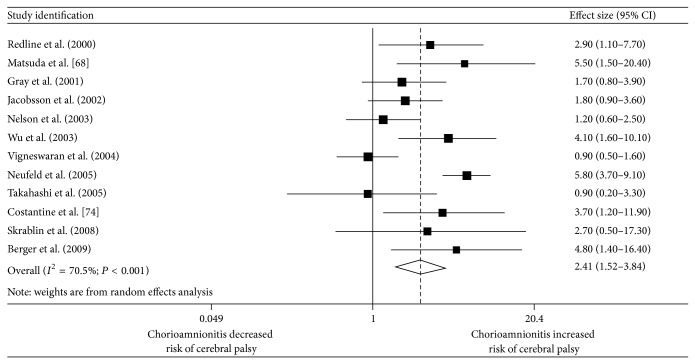
Meta-analysis of the association between clinical chorioamnionitis and cerebral palsy [40].

In 2000, Wu and Colford published a meta-analysis of 26 studies on the correlation between chorioamnionitis and infantile cerebral palsy [39]. Their analysis showed a significant correlation with a relative risk of 1.9 (95% CI 1.4–2.5). This data was confirmed by another meta-analysis published in 2010 [40] (Figure 4). Unfortunately, it was shown that the incidence of cerebral palsy could not be reduced by applying antibiotics as soon as chorioamnionitis had been diagnosed. Obviously the pathophysiological processes which led to the damage of the fetal brain were too advanced to be halted by means of therapeutic intervention. More efforts should therefore be undertaken in detecting ascending intrauterine infection very early in pregnancy. Hence, treatment of urinary tract infection by antibiotics has been shown to reduce the rate of preterm delivery [41]. Unfortunately, this effect could not be demonstrated for bacterial vaginosis [42, 43].

## 3. Prevention of Preterm Birth and Ascending Intrauterine Infections

Due to the fact that PIVH and PVL are complications which especially affect extreme preterm infants, both can be avoided by preventing the baby from being born preterm. Much evidence has shown that patients who have previously experienced a preterm birth or who develop shortening of the cervix to less than 25 mm before the 24th week of pregnancy benefit from the prescription of progesterone [44–48]. The latter group of patients should receive 200 mg of progesterone daily by vaginal suppositories, whereas women with a history of preterm birth can be treated either with a weekly intramuscular application of 250 mg 17-hydroxyprogesterone caproate or by means of a daily dose of 200 mg progesterone administered vaginally or 100 mg administered orally [44–49]. The use of a cervical cerclage in patients who have previously experienced a preterm birth and develop shortening of the cervix under 25 mm before the 24th week of pregnancy can also significantly reduce the likelihood of preterm birth [50, 51]. Interestingly, the outcome of these patients does not differ no matter whether they got a history indicated or a secondary cerclage. However, the latter procedure can help to avoid unnecessary surgical interventions [52].

Ascending intrauterine infections, which are often observed in patients at risk of preterm birth, seem to significantly increase the risk of fetal brain damage [30]. Bacterial vaginal infections should therefore be consistently treated during pregnancy to prevent the ascension of the infection to the unborn child. With this in mind, it is very interesting to note that progesterone has anti-inflammatory properties. Animal experiments have demonstrated a modulating influence on the gene-activation of COX-2, Connexin-43, TNF-a and IL-1 beta, and Toll-link receptors 2 and 4. The proteins associated with these receptors play a central role in the induction of preterm birth [53, 54]. If the infection has reached the intrauterine environment, it is necessary to consider inducing delivery immediately to prevent any further damage to the infant [39]. Unfortunately we are still lacking sound clinical parameters with which to make adequate medical decisions in this challenging situation.

## 4. Magnesium

If a preterm birth seems imminent, the infant's brain should be protected by means of the intravenous application of magnesium [55–57]. Within the last years many experimental studies have been published on the neuroprotective effects of magnesium. During acute cerebral ischemia large amounts of excitotoxic amino acids such as glutamate are released presynaptically. These neurotransmitters activate neuronal NMDA-receptors that operate calcium channels. As a consequence large amounts of calcium ions flow through these channels down an extreme extra-/intracellular concentration gradient, into the cell. Excessive increase in intracellular levels of calcium, so-called calcium overload, leads to cell damage through the activation of proteases, lipases, and endonucleases [58]. Magnesium ion gates the NMDA channels in a voltage-dependent manner and protects the brain from NMDA receptor-mediated injury [59, 60]. Moreover, magnesium suppresses cerebral convulsions and is a well known vasodilator [61, 62]. Both effects are known to be neuroprotective. Finally, magnesium has also been shown to decrease the release of nitric oxide and therefore reduce the postischemic production of oxygen radicals [63].

Magnesium is a substance which has been used for decades in the field of obstetrics as a prophylaxis for eclamptic seizures and tocolysis. In a case-control study which included infants weighing less than 1500 grams whose mothers were treated with magnesium [64] the authors established that children suffering from infantile cerebral palsy were less likely to have been exposed to magnesium sulfate than their healthy matched pairs and deduced from these findings that magnesium sulfate has a positive effect on very-low-birthweight infants. Several subsequent observational studies reported similar findings [65–74]. To address this open question, a series of controlled, randomized studies was initiated which included mothers who had been treated with MgSO_4_ for the purposes of fetal neuroprotection [75–78].

In August 2008, Rouse and colleagues published the results of the BEAM (Beneficial Effects of Antenatal Magnesium Sulfate) study, conducted by the Maternal-Fetal Medicine Units Network [79]. The primary outcome of this high quality study on the incidence of infantile cerebral palsy among children whose mothers had been treated with MgSO_4_ was the combined occurrence of infantile cerebral palsy (of serious or medium severity) or death. No significant difference in the combined risk levels was identified between the therapy and control groups. When the combined results were disaggregated, given similar mortality rates, a significantly lower rate of infantile cerebral palsy was identified among children whose mothers had been treated with magnesium sulfate (1.9 versus 3.5%). Rouse and colleagues concluded from these results that the application of MgSO_4_ leads to a reduction in the incidence of cerebral palsy among very preterm infants [79].

In 2009, a Cochrane Review was published on the topic [55]. The five prospective-randomized studies it covered, which were published between 2002 and 2008, included a total of 6145 children. The effect of magnesium sulfate as a neuroprotective agent was tested on patients at risk of preterm birth before the end of the 37th week of pregnancy. The studies found a significant reduction in the incidence of infantile cerebral palsy (relative risk 0.68; 95% confidence interval 0.54 to 0.87), as well as in the incidence of gross motor skill dysfunction (relative risk 0.61; 95% confidence interval 0.44 to 0.85) among children whose mothers had been treated with magnesium sulfate (Table 1). Doyle and coworkers reevaluated the children from the ACTOMgSO_4_ trial, one of the five above mentioned prospective studies, at school age. The effects of magnesium on the rate of cerebral palsy and abnormal motor function were no longer evident at this time. Possibly, additional therapeutic interventions in the control group may have improved the health status of these children [80].

The number of women at risk of preterm birth who need to be treated with MgSO_4_ to prevent one case of infantile cerebral palsy (number needed to treat (NNT)) is dependent on the week of pregnancy in which the birth occurs; it is 52 before the 34th week of pregnancy [81] and 29 before the 28th week of pregnancy [82]. In the USA around 2000 cases of infantile cerebral palsy are reported annually. If all women who gave birth before the 34th week of pregnancy were treated with magnesium sulfate, around 660 children per year could be spared from infantile cerebral palsy. The cost of preventing these cases would amount to around $10,291 USD annually [81].

The most commonly reported maternal side-effects of systematic magnesium therapy include thrush, sweating, nausea, vomiting, or skin irritation at the injection site. In addition, a 50% increase in the risk of hypotension and tachycardia was reported (number needed to harm (NNH): 28–30). A higher rate of serious complications such as maternal mortality, cardiac or respiratory arrest, pulmonary edema, respiratory depression, serious postpartal hemorrhage, or increased rate of cesarean sections was not identified.

In the various studies, differing amounts of magnesium were given to patients. The levels ranged from 4 g to almost 50 g of MgSO_4_. A statistically significant effect was first apparent above a moderate dosage of 4 g magnesium sulfate. There was no significant difference between the placebo group and children whose mothers received a lower dosage [81]. The total dosage of magnesium administered should be taken into consideration, as controversial results reported by the Mittendorf study are likely to be explained by the high magnesium dosages administered (up to 500 g) [83].

The treatment should begin with a bolus injection of 4–6 g within 30 minutes, followed by maintenance doses of 1-2 g/h for 12 hours. The aim of this procedure is to double the magnesium level in the mother's serum. If birth does not occur within 12 hours, the administration of magnesium can be restarted at a later point in time if preterm birth again appears imminent.

In an Australian perinatal center, Ow and colleagues investigated the rate of women which can be administered magnesium intravenously under clinical conditions for the purpose of neuroprotection in the event of an imminent preterm birth [84]. Out of 330 women at risk of preterm birth, 132 were given magnesium (132/330, 40%). A total of 74% of all women (142/191) were administered magnesium prior to a preterm birth before the 32nd week of pregnancy.

The administration of high dosages of magnesium in the event of an imminent preterm birth leads to a reduction in the rate of infantile cerebral palsy and gross motor skill dysfunction [56, 85].

## 5. Delayed Clamping of the Umbilical Cord 

An infant's blood volume at birth can be significantly influenced by the time at which the umbilical cord is clamped [86]. In 1988 Hofmeyr and colleagues published a randomized study investigating the outcome among preterm infants dependent upon the time blood flow in the umbilical cord was interrupted [87]. When the umbilical cord was clamped one minute after birth, the brain hemorrhage rate was 35%, compared to 77% when it was clamped immediately [87]. This effect is believed to be caused by the reduced risk of hypoperfusion and improved oxygen delivery to the brain [88]. Delayed clamping of the umbilical cord could also lead to an increase in the concentration of coagulation factors and in the number of stem cells, which have been shown to have neuroprotective effects in animal experiments [89, 90].

Several studies published since 1980 have shown that delayed clamping of the umbilical cord can reduce the need for blood and fluid transfusion, as well as the rate of brain hemorrhages and sepsis among preterm infants [87, 91–94]. However, delayed clamping of the umbilical cord has also been associated with polycythaemia, hyperbilirubinemia, and an increased need for phototherapy [91–94]. To clarify these issues, a prospective randomized study was initiated in 2006. Seventy-two women who experienced a preterm birth before the 32nd week of pregnancy were divided into two groups in which the umbilical cord was clamped either early or late (30–45 s after birth). A significant reduction in the rate of brain hemorrhages and sepsis among the infants whose umbilical cords were clamped later was observed. Other variables including bilirubin and the amount of blood transfused were not affected [94] (Table 2). On the basis of this data, the ACOG recommends delayed clamping of the umbilical cord among all preterm infants born before the 32nd week of pregnancy [95]. The infant should remain at the level of the placenta during this time. The incidence of brain hemorrhage can thus be reduced by up to 50%. It is likely that repeated milking the umbilical cord (four times) leads to similar results [96].

## 6. Experimental Approaches

In order to understand the effect of several other treatment measures which have been predominantly tested in animal experiments, it is important to comprehend the pathophysiological processes occurring during and after an injury. The initial damage caused by an injury is normally the result of insufficient metabolic supply. This leads to a loss of membrane potential, a massive release of excitatory neurotransmitters, and a very strong influx of calcium, which in turn activates proteases, endonucleases, and lipase and thus induces successive cell death [58]. However, significant cell damage can also occur in the early recovery phase, although oxidative phosphorylation has increased [97]. Electroencephalogram is normally suppressed and the cerebral blood flow is reduced in this phase, but oxygenation of the brain usually remains within physiological limits [98, 99]. After approximately 6–15 hours, seizures occur, along with a renewed alteration of the mitochondrial metabolism, cell edema, and subsequent secondary cerebral lesions [97, 98, 100]. Impairment of subsequent neurologic development is strongly affected by this phase [101]. Such secondary damage is often followed by a phase involving tertiary damage as a result of a lack of growth factors, synaptic input, and immigrating neuronal and glial stem cells [102–105].

## 7. Stem Cells 

This is the point at which therapy with so-called stem cells becomes relevant. Stem cells can be obtained from many different types of tissue. Depending on their origin, they are referred to as neuronal, mesenchymal, or hematopoietic stem cells, and so forth. Mesenchymal stem cells are currently considered to have the most potential for clinical applications. They can be grown easily from bone marrow and from extraembryonic tissue such as the placenta, Wharton's jelly, and umbilical cord stroma [106–108].

**Figure 5:**
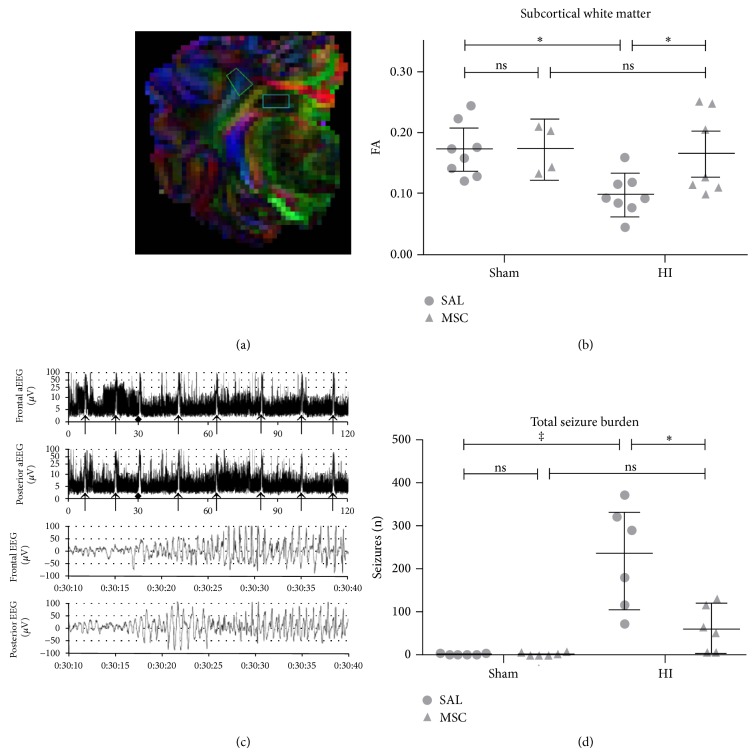
Hypoxic-ischemic brain damage was induced in fetal sheep on the 105th day of gestation (term is at 150 days) by occluding the umbilical cord for 25 minutes. One hour after the injury had been inflicted, the animals in study group were given i.v. 3.5 × 10^6^ mesenchymal stem cells (MSCs), whereas those in the control group received saline (SAL). In sham-operated animals (Sham) occlusion was not carried out. (a) Diffusion Tensor Images (DTI) measured using magnetic resonance tomography (MRI) presented as fractional anisotropy (FA). Regions of interest SCWM (subcortical white matter) and hippocampus. (b) Mesenchymal stem cells reduce brain damage in the SCWM, measured as FA. Means ± 95% CI (^*^ *P* < 0.05) are depicted. Dots show each measurement per animal. (c) Representative image of an aEEG trace from an animal of the control group with hypoxic-ischemic brain damage. The arrows mark seizures. (d) The administration of MSC significantly reduces the number of seizures (*n*) (^*^ *P* < 0.05; ^‡^ *P* < 0.01) [112].

**Figure 6:**
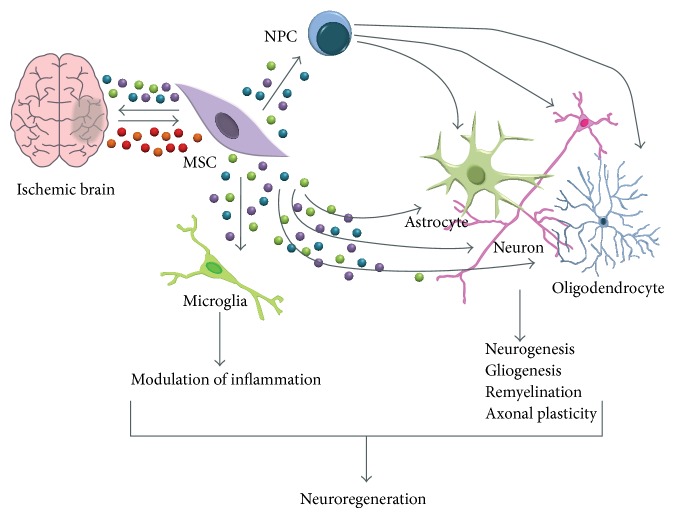
Neuroregeneration using mesenchymal stem cells (MSCs) following neonatal hypoxia-ischemia. After transplantation into an ischemically damaged area of the brain mesenchymal stem cells respond to the environment by producing growth and differentiation factors. These factors stimulate proliferation and differentiation of neural stem cells (NPCs) and stimulate repair processes. Upon transplantation of MSCs, microglia are activated and modulate the inflammatory response of the brain contributing to neurorepair [113].

Originally it was believed that the applied stem cells multiplied in the damaged region, where they differentiated and replaced the destroyed tissue. However, it quickly became clear that this could not be the way the neuroprotective mechanism worked. The low number of stem cells growing and the insufficient rate at which they differentiate in no way explained the significant neuronal improvements observed. Recent animal experiments have shown that the application of stem cells leads to a significantly improved outcome following hypoxic-ischemic damage [109–111]. This neuroprotective effect has recently also been demonstrated in preterm sheep fetuses [112] (Figure 5). The stem cells applied to damaged tissue appear to release numerous factors in the damaged area which induce the formation and migration of neuronal stem cells, encourage the expansion of dendrites and axons, and suppress postischemic inflammation [111, 113] (Figure 6).

Via transmitters, mesenchymal stem cells modulate numerous signaling cascades during apoptosis, neurogenesis, angiogenesis, and synaptogenesis. Increased expressions of fibroblast growth factor-2, epidermal growth factor, glial cell line-derived neurotrophic factor, and sonic hedgehog have been observed [114]. These factors play a central role in the proliferation of progenitor cells, as well as in neurogenesis and cell differentiation [115–120] (Figure 6). Mesenchymal stem cells stimulate the proliferation of progenitor cells in the dentate gyrus. These progenitor cells move into the damaged area and differentiate under the influence of mesenchymal stem cells into astrocytes, oligodendrocytes, and neurons [110, 121]. Additionally, stem cells induce the formation of neuropilin-1 and 2, neuregulin-1, and EphrinB2, messengers which play an important role in the regulation of axonal growth, synapse formation, and the integration of the neuronal network [114, 122]. Mesenchymal stem cells support the proliferation and differentiation of oligodendrocyte-progenitor cells and thus the myelinisation of the newly formed axons [110, 123, 124]. Additionally, mesenchymal stem cells appear to counteract glial scar formation, which hinders the migration of axons and dendrites [125, 126] (Figure 6).

Postischemic inflammation is most commonly caused by microglia, macrophages of the central nervous system, which originate in bone marrow and migrate to the brain during development, where they then differentiate into microglia. When brain damage occurs, these local microglia are activated, and monocytes from peripheral blood also migrate to the trauma site [127, 128]. These so-called M1 microglia release proinflammatory cytokines, oxygen-based free radicals, and neurotoxins and further damage the altered tissue. There is an alternative pathway for the activation of microglia (M2), which has neuroprotective effects and leads to the release of IL-10, insulin growth factor-1, transforming growth factor-*β*, and other immunomodulating factors [128–130]. The application of mesenchymal stem cells reduces the number of classically activated microglia (M1) and thus also inhibits the release of proinflammatory cytokines [112, 114, 124]. In contrast, the M2 cascade is activated with synthesis of growth factors supporting the regeneration of damaged tissue [131, 132] (Figure 6).

To what extent effects caused by mesenchymal stem cells can also be instigated by exosomes remains to be seen. Exosomes are small membrane vesicles (70–120 nm) which contain lipids, proteins, and RNA and which are secreted by various types of cells. Exosomes obtained from mesenchymal stem cells have been shown to reduce the extent of myocardial damage following ischemia in experiments with adult mice [133].

## 8. Estradiol and Progesterone

The steroid hormones estradiol and progesterone play a critical role in the growth, differentiation, and function of the reproductive system. However, the peripheral and central nervous system is also affected by these hormones, as shown by the ubiquitous distribution of the relevant receptors [134–137]. Estradiol induces axonal and dendritic growth and promotes the development of synapses as well as the integration of the cerebral cortex [138].

**Figure 7:**
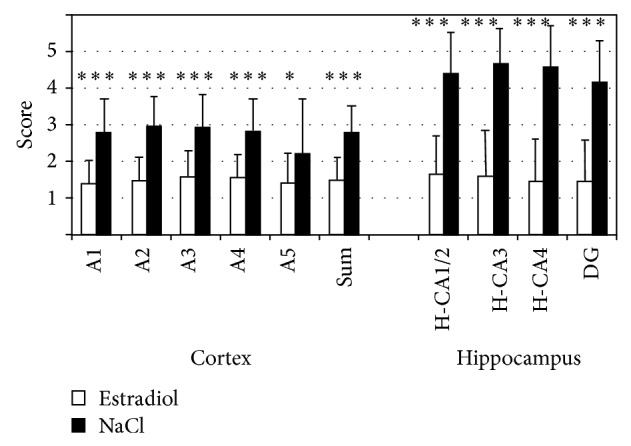
Neuronal cell damage in the cerebral cortex (A1–A5) and hippocampus (CA 1/2, CA3, DG) in neonatal rats following hypoxia-ischemia. The animals in the study group were treated with estradiol both before and after injury, whereas animals of the control group received NaCl. Neuronal cell damage was significantly reduced in the cerebral cortex as well as in the hippocampus after application of estradiol. Neuronal cell damage was evaluated using a scoring system (1 = 0%–4%; 2 = 5%–49%; 3 = 50%–94%; 4 = 95%–99%; 5 = 100% damaged neurons). Means ± SD (^*^ *P* < 0.05; ^***^ *P* < 0.001) [143].

In the third trimester of pregnancy, the maternal serum concentration of estradiol and progesterone increases significantly and can reach up to 100 times its original level [139, 140]. Preterm infants are removed abruptly from this environment. In animal experiments estradiol has been shown to protect the immature fetal brain from hypoxic-ischemic lesions [141–143] (Figure 7). Progesterone has also been shown to have neuroprotective effects [144]. It therefore makes sense to treat preterm infants with estradiol and progesterone after birth [145]. Unfortunately, there is currently a lack of sufficient clinical data to support such treatment [146].

The neuroprotective effects of estradiol can be imparted receptor-dependent (genomic and nongenomic) or receptor-independent [147–149]. The estrogen-receptor independent effects are the result of the direct antioxidative properties of estradiol and of the interaction with potential binding sites on the neuronal membrane receptors [147]. These membrane receptors can modulate the neurotransmission and excitability of the neuronal membrane [150, 151]. The classic receptor-mediated effects on neuronal gene transcription cause the growth of axons and dendrites, the creation of synapses, the expression of neurotropic factors, and increased acetylcholine synthesis. In the central nervous system, the estrogen receptor subtypes *α* und *β* have been shown to be differently distributed and regulated [134, 135]. Both receptors have the same affinity for estradiol [152], but differing levels of affinity for “estrogen-response-elements,” and have therefore demonstrated partially different gene activation patterns [153]. Two effects of the estradiol receptor-*α* are responsible for the antiapoptotic properties. The activation of the receptor leads to a rapid induction of the insulin-like growth factor 1 (IGF-1) receptor pathway and the associated signal cascade [154]. IGF-1 has been shown to have neuroprotective capacity [155]. In addition, 17*β*-estradiol inhibits the caspase-pathway which plays a key role in apoptosis [156].

The neuroprotective effects of progesterone are also mediated by various mechanisms. Progesterone reduces postischemic cellular edema by maintaining the integrity of the blood-brain barrier. Increased expressions of claudin5 and occludin1 have been observed in connection with this process, both of which are proteins that play an important role in the creation of tight junctions. In contrast, it has been shown that the expression of MMP-3 and MMP-9 is reduced. The latter is involved in extracellular tissue degradation [157, 158]. Additionally, progesterone inhibits postischemic apoptosis and induces the release of the growth factor BNDF, as has been demonstrated by investigations using the TUNEL assay and caspase 3 [159]. Progesterone suppresses postischemic inflammation by reducing the expression of IL-1*β*, TNF-*α*, IL 6, COX-2, and ICAM-1 [146, 149–151]. It also reduces the expression of TGF-*β*2, VCAM-1, CD68, and Iba1 [160–162], factors which play a role in postischemic inflammation. The inducible form of NO-synthase is inhibited by progesterone [163], while the levels of superoxide dismutase, catalase, and glutathione peroxidase in tissue are increased [164]. The administration of progesterone also leads to the increased release of GAP43 and synaptophysin, both of which are markers for synaptogenesis [165]. However, one study including experiments with rats showed an increase in hypoxic-ischemic brain damage following the administration of progesterone on the 7th and 14th day of life, but not on the 21st day [166].

Many more neuroprotective strategies have been investigated in animal experiments. However, a detailed discussion of all of these strategies is outside the scope of this review. We would like to invite interested readers to refer to the relevant literature.

## Figures and Tables

**Table 1:** Magnesium sulfate for neuroprotection.

	Magnesium (*N*)	Control (*N*)	RR, 95% CI
Cerebral palsy	104/3052	154/3093	0.68 (0.54–0.87), *P* = 0.002
Gross motor skill dysfunction	57/2967	94/3013	0.61 (0.44–0.85), *P* = 0.003
Infant mortality	443/3052	430/3093	1.04 (0.92–1.17)

Antenatal administration of magnesium sulfate significantly reduced the rate of cerebral palsy and gross motor skill dysfunction among preterm infants. The infant mortality rate remained unchanged [55].

**Table 2:** Neuroprotection by delayed clamping of the umbilical cord.

	ICC (*N* = 36), *N* (%)	DCC (*N* = 36), *N* (%)	*P*	Odds ratio	95% CI
IVH					
Total	**13 (36)**	**5 (14)**	**0.03**	**3.5**	**1.1–11**
Grade 1	4 (11)	3 (8)			
Grade 2	8 (22)	2 (6)			
Grade 4	1 (3)	0 (0)			
Sepsis	8 (22)	1 (3)	0.03	0.1	0.01–0.84

Among infants born before the 32nd week of pregnancy, delayed clamping of the umbilical cord (DCC: delayed cord clamping 30–45 s) reduced the rate of intraventricular brain hemorrhage (IHV) and neonatal sepsis significantly when compared to immediate severance of the cord (ICC: immediate cord clamping 5–10 s) [94].

## Condensation

Clinical and experimental strategies to protect the immature brain from ischemic and/or infectious injury are reviewed.
